# Does a Passive Unilateral Lower Limb Exoskeleton Affect Human Static and Dynamic Balance Control?

**DOI:** 10.3389/fspor.2019.00022

**Published:** 2019-09-20

**Authors:** Steffen Ringhof, Isabel Patzer, Jonas Beil, Tamim Asfour, Thorsten Stein

**Affiliations:** ^1^Department of Sport and Sport Science, University of Freiburg, Freiburg, Germany; ^2^BioMotion Center, Institute of Sports and Sports Science, Karlsruhe Institute of Technology (KIT), Karlsruhe, Germany; ^3^High Performance Humanoid Technologies, Institute for Anthropomatics and Robotics, Karlsruhe Institute of Technology (KIT), Karlsruhe, Germany

**Keywords:** exoskeleton, human-technology-interaction, biomechanics, balance, motor control, posturography, braces

## Abstract

Exoskeletons are wearable devices closely coupled to the human, which can interact with the musculoskeletal system, e. g., to augment physical and functional capabilities. A main prerequisite for the development and application of exoskeletons is to investigate the human-exoskeleton interaction, particularly in terms of potential inferences with human motor control. Therefore, the purpose of the present study was to investigate whether a passive unilateral lower limb exoskeleton has an impact on static and dynamic reactive balance control. Eleven healthy subjects (22.9 ± 2.5 years, five females) volunteered for this study and performed three different balance tasks: bipedal standing, single-leg standing, and platform perturbations in single-leg standing. All the balance tasks were conducted with and without a passive unilateral lower limb exoskeleton, while force plates and a motion capture system were used to capture the center of pressure mean sway velocity and the time to stabilization, respectively. Dependent *t*-tests were separately run for both static balance tests, and a repeated-measure analysis of variance with factors exoskeleton and direction of perturbation was calculated for the dynamic reactive balance task. The exoskeleton did not significantly influence postural sway in bipedal stance. However, in single-leg stance, the mediolateral mean sway velocity of the center of pressure was significantly shorter for the exoskeleton condition. For the dynamic reactive balance task, the participants tended to regain stability less quickly with the exoskeleton, as indicated by a large effect size and longer time to stabilization for all directions of perturbation. In summary, the study showed that the exoskeleton provided some assistive support under static conditions, which however may disappear when sufficient stability is available (bipedal stance). Besides, the exoskeleton tended to impair dynamic reactive balance, potentially by impeding adequate compensatory adjustments. These are important findings with strong implications for the future design and application of exoskeletons, emphasizing the significance of taking into account the mechanisms of human motor control.

## Introduction

Exoskeletons are wearable devices closely coupled to the human, which interact with the musculoskeletal system by applying assistive forces to the limb or restricting the user's mobility. In recent years, the development and application of exoskeletons has experienced increasing popularity. Especially in neuro-rehabilitation, lower limb exoskeletons (LLExo) have gained considerable interests, since they can help patients with paralysis or spinal cord injury to stand and walk again (Schwartz and Meiner, 2015; Chen et al., 2018). Another important field of application relates to the augmentation of human capabilities. Herein, exoskeletons are commonly used to reduce the stress and metabolic cost during physically demanding tasks (Zoss et al., 2006; Bosch et al., 2016; Galle et al., 2017; Huysamen et al., 2018) or to prevent falls in the elderly (Giovacchini et al., 2015; Verrusio et al., 2017). Furthermore, as physical and functional losses increase with the aging of the population (e.g., sensory loss, slower reaction time, decreased limb muscle force and power, reduced oxygen consumption), just recently Grimmer et al. (2019) emphasized the potential of LLExos to compensate for such losses.

A main prerequisite for the design and application of LLExos is to consider the anatomy and the biomechanics of the human musculoskeletal system. Furthermore, in order to relieve the stress on the human body and to augment human movements, it is essential that LLExos are kinematically compatible, can be controlled intuitively and have no negative effects on the user's motor functions. Hence, from a practical point of view, there is a need for LLExos that are able to mechanically guide and assist the user, while still allowing for the intended extent of movements as well as the maintenance of balance (Grimmer et al., 2019). Bearing this in mind, it is of particular importance to investigate the human-exoskeleton interaction in terms of potential inferences with human motor control mechanisms. Especially the assessment of human balance is critical to evaluate the compatibility and operator safety of LLExos (Mummolo et al., 2018).

In general, the term balance describes the dynamics of body posture to ensure stability and prevent falling (Winter, 1995). Hence, the control of balance is crucial not only for many sports but also for most activities of daily living (Woollacott et al., 1986). The literature commonly distinguishes between static and dynamic conditions of balance. While dynamic stability is considered the maintenance or recovery of balance in response to internal or external disturbances (Horak et al., 1997), static stability relates to balance control under unperturbed conditions such as during quiet standing (Macpherson and Horak, 2013). However, as the evolution to bipedalism forces humans to control a high center of mass over a small base of support, the central nervous systems even under static conditions is continuously required to apply suitable strategies for dynamically controlling balance (Woollacott et al., 1986; Monaco et al., 2017).

Given the high relevance of balance control from scientific and socioeconomic perspectives, the assessment of balance under static and dynamic conditions is an important issue when assessing human-exoskeleton interactions. Therefore, the question remains as to whether LLExos may contribute to an improved postural stability, in terms of increased mechanical support, or may rather impair postural stability, in terms of impeding relevant motor adjustments that would be necessary to properly maintain or restore balance.

Despite its relevance, to the best of our knowledge there is only two studies available that have investigated the influence of LLExo on human balance control (Schiffman et al., 2008; Emmens et al., 2018). Emmens et al. (2018) showed that a powered ankle-foot orthosis with a body sway based controlled was able to assist the subjects in maintaining balance when counteracting anteroposterior perturbations, which was accompanied by decreased soleus and tibialis anterior muscle activity. The study by Schiffman et al. (2008) revealed that a passive LLExo reduced postural sway in 10 US army men, irrespective of whether they were carrying different loads. Therefore, the authors suggested that the exoskeleton structure may have provided a bracing effect on the subjects' body, increasing their postural stability. Other studies dealing with the augmentation or interference of human motor control mainly have been focusing on the impact of LLExos on gait adaptations. Herein, it was found that both active and passive LLExos decreased the participants' gait speed and increased the metabolic cost of walking when compared to a control condition (Gregorczyk et al., 2010). Nonetheless, yet there is no study that has investigated the impact of a passive LLExo on dynamic balance control.

Given the exceptional scientific and medical relevance of developing high performance exoskeletons along with a lack of studies concerning the interference of passive LLExos with human balance control, our study addressed two main research questions. First, we investigated whether wearing a passive unilateral LLExo has an impact on balance control during quiet standing (static balance). Secondly, we investigated whether the exoskeleton does influence dynamic stability when compensating a random perturbation applied to an unstable support surface (dynamic reactive balance). We hypothesized that our LLExo would provide passive mechanical support that improves static stability, whereas dynamic stability would be decreased due to restrictions of compensatory lower limb movements.

## Materials and Methods

### Participants

Eleven healthy subjects (22.9 ± 2.5 years), six males and five females, volunteered for this study. Due to the constraints of the exoskeleton structure, the sample of the subjects had to be very homogeneous. Specifically, taking into account the solid parts of the exoskeleton, it was only possible for people of a certain height and weight to wear the exoskeleton. The length of the shank had to be between 40.0 and 42.0 cm and the shoe size had to be around 42.5 (EUR size). In the end, the height of the subjects was 1.75 ± 0.04 m and the body mass 73.1 ± 5.3 kg. Further exclusion criteria comprised any musculoskeletal, neurological or cardiovascular diseases that could have affected the participant's ability to perform the experiments.

All participants were informed regarding the nature and aim of the study and gave their written informed consent before participating in the study. The experimental protocol was approved by the ethics committee of the Karlsruhe Institute of Technology.

### Experimental Design

Each participant had to perform three balance tasks: quiet standing on a force plate in bipedal stance, quiet standing on a force plate in single-leg stance, and compensating a perturbation applied to a swinging platform in single-leg stance. These tests are frequently applied to investigate human balance control (e.g., Winter et al., 1990; Era et al., 2006; Petró et al., 2017) as well as to evaluate balance training interventions (e.g., Giboin et al., 2015; Krause et al., 2018; Ringhof et al., 2018). In general, quiet standing on the force plate is considered static steady state balance, whereas the compensation of the platform perturbation is considered dynamic reactive balance.

The participants performed all the balance tasks with and without a passive unilateral LLExo. The order was counterbalanced, which means that half of the subjects (in our case six subjects) started the experiments with the exoskeleton [EXO], whereas the other half of the subjects (in our case five subjects) started first performing the tasks without the exoskeleton [No-EXO]. This experimental design compensates for potential learning and fatigue effects.

The limb chosen for single-leg standing was therefore always the left leg on which the test persons had worn the exoskeleton.

### Exoskeleton

The passive LLExo used in the present study is a modified version of the system described in Beil et al. (2018). This system has been developed for human motion classification purposes and uses Hidden Markov Models to provide an online classification of previously defined motion patterns based on seven 3D force sensors and three inertial measurement units that are incorporated into the LLExo. This information is to be used to improve the user acceptance as well as the actuator control in the active lower limb exoskeleton on which our work is originally based (Beil et al., 2015). However, as this study was conducted to get a fundamental knowledge about the impact of exoskeleton on the human body and balance control, a passive and minimal setup without the latter techniques and machine learning algorithms was chosen.

**Figure 1:**
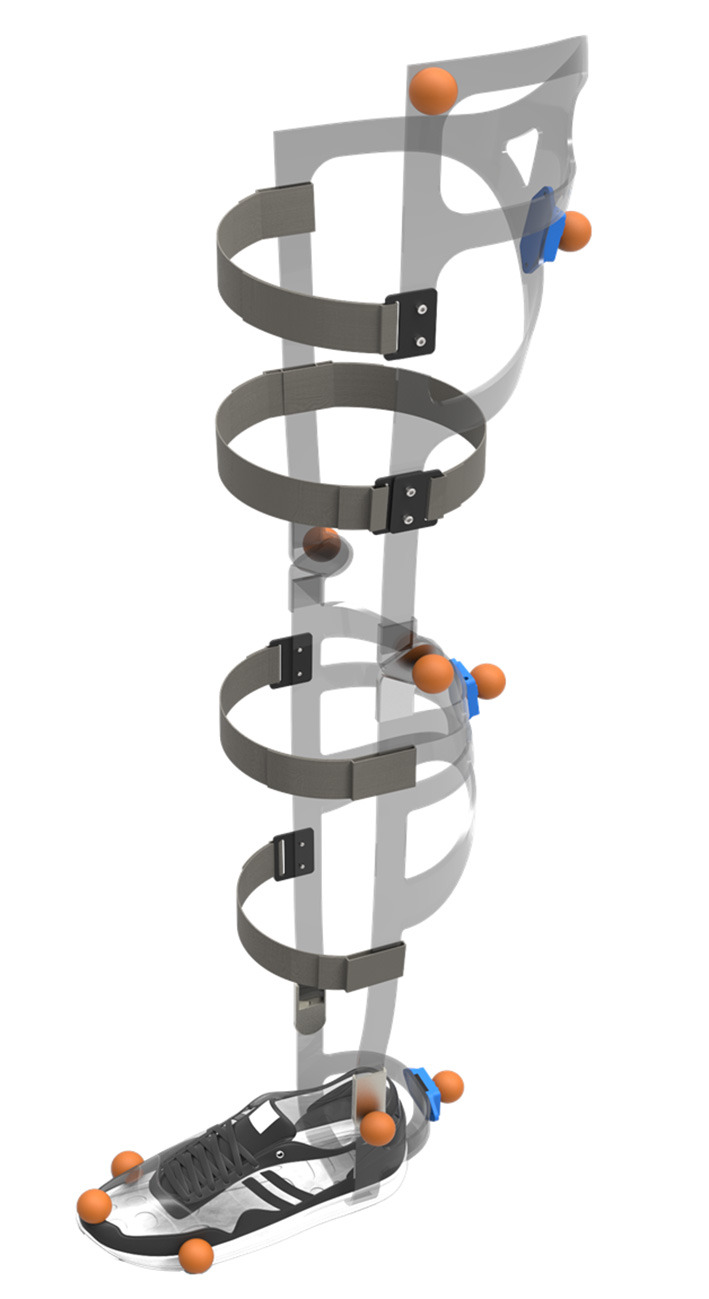
Schematic illustration of the exoskeleton. The exoskeleton consists of three aluminum frame parts (light gray) connected by one degree of freedom revolute joints at the knee and ankle. Subjects are secured to the device by orthotic straps on the thigh and shank (dark gray) and a sports shoe at the foot. Reflective markers (orange) and IMUs (blue) are attached to the frames of the exoskeleton to track the motion of the system (not used in the present study).

In more detail, the exoskeleton used in the present study consists of three basic aluminum frame parts for the thigh, the shank and the foot of the left limb (Figure 1). These parts are connected by one degree of freedom (DoF) orthotic revolute joints (Otto Bock, 17B47 = 20/17B57 = 20) at the knee and ankle, permitting joint motions in the sagittal plane only. Using soft aluminum (EN-AW 5083) allowed for the adjustment of the frames to the inter-subject leg characteristics. By using this kind of material, furthermore, a slight compensation of the missing DoF at the ankle and knee joint is provided. Subjects were secured to the device by two orthotic Velcro straps on the thigh and shank, respectively, as well as a sports shoe (Adidas Duramo 7) at the foot. Since segment length adjustments of the exoskeleton frames was not possible to this point, only subjects with fitting lower limb segment length and shoe size were allowed to participate in this study.

### Experimental Procedure

The participants were tested individually in one session. After signing the consent form, participants warmed up and familiarized themselves with the tasks and the exoskeleton. Due to the crossover design, two different warm-up protocols were used. Prior to the No-EXO condition, the participants warmed-up on a treadmill for 5 min at 5 km/h. Prior to the EXO condition, the warm-up included a 2 min' walk in the exoskeleton at a self-selected speed, a 5 min' walk on the treadmill at 5 km/h and a final 2 min' walk at a self-selected speed.

Measurements began with standardized verbal instructions and one familiarization trial for each task. Then three valid trials were recorded for each task and experimental condition [EXO, No-EXO]. The balance tasks were performed in the following order.

#### Bipedal Static Balance

**Figure 2:**
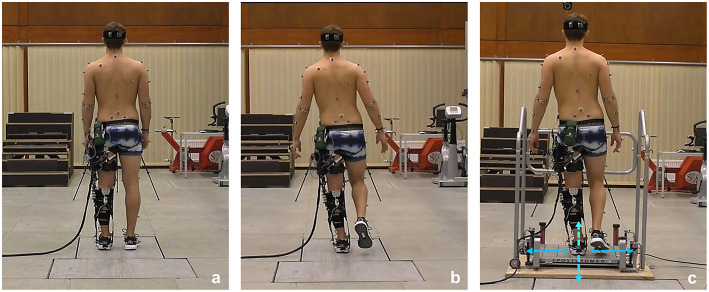
Balance tests conducted in this study: **(a)** bipedal stance on the force plate; **(b)** single-leg stance on the force plate; **(c)** single-leg stance on the Posturomed with directions of perturbation applied to the platform.

Static balance in bipedal stance was conducted with the feet hip-width apart, the eyes open, and arm hanging on the side (Figure 2a). The participants were asked to remain as stable as possible and to focus on a target positioned at eye-level at a distance of 4 m. Data were acquired for 30 s while standing on a force plate (AMTI, model BP600900; Advanced Mechanical Technology, Watertown, USA). The force plate was embedded in the ground and measured 3D ground reaction forces at 1,000 Hz.

#### Single-Leg Static Balance

The experimental conditions for the single-leg stance were essentially the same as for the bipedal stance. Hence, the participants were instructed to remain as stable as possible while eyes focusing on the target and arms hanging at their sides. However, they were asked to elevate their right leg and to refrain from touching the ground or the standing leg (Figure 2b). As for the bipedal static balance task, data were acquired for 30 s by using the force plate.

#### Dynamic Reactive Balance

To investigate dynamic reactive balance, we assessed the participants' capacity to respond to perturbations of an unstable platform. The participants were asked to stand on a Posturomed (Haider Bioswing GmbH, Pullenreuth, Germany) adopting a single-leg stance, with hands hanging at their sides and eyes focusing on the target (Figure 2c). The Posturomed consists of a multi-axial free-swinging platform (0.6 × 0.6 m) that is connected to a metallic frame by dampening elements allowing for free damped oscillations of the platform in all directions of the transverse plane.

By use of a custom-made release system, the platform was displaced horizontally up to 5 cm away from its center position (Ringhof et al., 2018). The perturbation was applied randomly in an anterior, posterior, medial, or lateral direction by electrical stimuli inducing mechanical pushing against the metallic frame structure below the platform. The perturbation lasted about 500 ms, whereupon the platform was released in the opposite direction. The participants' task was to compensate for these perturbations and thereby to stabilize the platform as quickly as possible (Ringhof and Stein, 2018).

To assess the participants' performance, four reflective markers (diameter 14 mm) were attached to the platform. Their displacements were recorded by 13 infrared cameras at 200 Hz (Vicon MX cameras, Vicon Motion Systems; Oxford Metrics Group, Oxford, UK). The marker placement along with the time continuous recording allowed the center of the Posturomed and its displacements to be defined.

### Data Analysis

All data were pre-processed using Vicon Nexus 1.8.5 and were then analyzed using MATLAB R2016a (The MathWorks, Natick, MA, USA). The data were initially filtered by fourth-order Butterworth low-pass filters, with cut-off frequencies of 10 Hz for CoP displacements and 20 Hz for kinematics of the Posturomed. Then, static and dynamic reactive balance was quantified as follows.

For both static balance tasks, the mean sway velocity as well as the mean frequency of the center of pressure (CoP) time signal were computed (Pinsault and Vuillerme, 2009). In general, the CoP mean velocity is considered to be a valid and reliable outcome measure to represent the ability and amount of neuromuscular activity to control balance (Ruhe et al., 2010). The mean frequency provides a view of the frequency content of the CoP signal, with higher frequencies being indicative of faster and smaller postural adjustments as well as higher stiffness around the ankle joint (Warnica et al., 2014). Both variables were decomposed into anteroposterior and mediolateral components in order to get more detailed information about the directional impact of the exoskeleton on static balance control.

Dynamic reactive balance on the Posturomed was quantified by the time to stabilization (TTS), which indicates the time the participants needed to compensate for the perturbations applied to the platform. The perturbations were considered compensated when the platform deviations from its center position were < ±2 mm for at least 500 ms (Giboin et al., 2015).

For each balance test and experimental condition [EXO, No-EXO], all three valid trials were included in the analysis, based on which mean values were calculated.

### Statistics

Statistical analyses were performed using IBM SPSS Statistics 24.0 (International Business Machines Corporation; Armonk, USA). After confirmation of normality and sphericity of data distribution, dependent *t*-tests were separately run for both static balance tests—bipedal stance and single-leg stance—to compare the participants' balance measures between both experimental conditions [EXO, No-EXO].

For the dynamic reactive balance task, a repeated-measures analysis of variance (ANOVA) with factors exoskeleton [EXO, No-EXO] and direction of perturbation [anterior, posterior, medial, lateral] was calculated. In case of statistical significances or relevant effect sizes, *post-hoc t*-tests were used for pairwise comparisons.

All data are presented as mean values and 95% confidence intervals (95% CI). Statistical differences are reported by their level of significance. Additionally, effect sizes are indicated using Cohen's *d* (small effect: 0.20 ≤ *d* < 0.50; medium effect: 0.50 ≤ *d* < 0.80; large effect: *d* ≥ 0.80) and partial eta squared (small effect: 0.01 ≤ $n_{p}^{2}$ <0.06; medium effect: 0.06 ≤ $n_{p}^{2}$ <0.14; large effect: $n_{p}^{2}$ ≥ 0.14) for *t*-tests and ANOVA, respectively (Cohen, 1988; Richardson, 2011). The level of significance for all statistical tests was set a priori to *p* = 0.05.

## Results

The participants' performance in the different balance tests with and without the exoskeleton is shown in Table 1.

**Table 1:** Participants' performance in the different balance tests with (EXO) and without the exoskeleton (No-EXO).

	**EXO**	**No-EXO**	**Difference**	***p-*value (Cohen's *d*)**
**Bipedal static balance**				
CoP velocity AP (mm/s)	6.49 (0.67)	6.18 (0.74)	0.31	0.456 (0.23)
CoP velocity ML (mm/s)	4.14 (0.52)	3.94 (0.59)	0.20	0.408 (0.16)
Mean frequency AP (Hz)	0.20 (0.04)	0.19 (0.03)	0.02	0.297 (0.24)
Mean frequency ML (Hz)	0.35 (0.06)	0.40 (0.05)	−0.04	0.229 (0.44)
**Single-leg static balance**				
CoP velocity AP (mm/s)	23.01 (3.15)	22.38 (3.30)	0.63	0.565 (0.18)
CoP velocity ML (mm/s)	21.80 (2.90)	26.10 (4.16)	−4.29	0.018 (1.13)*
Mean frequency AP (Hz)	0.33 (0.07)	0.32 (0.06)	0.01	0.865 (0.05)
Mean frequency ML (Hz)	0.57 (0.09)	0.68 (0.10)	−0.11	0.003 (1.23)*
**Dynamic reactive balance**				
TTS anterior (s)	1.67 (0.54)	1.29 (0.23)	0.38	0.126 (0.46)
TTS posterior (s)	1.76 (0.45)	1.44 (0.23)	0.32	0.061 (0.35)
TTS lateral (s)	2.37 (0.92)	1.84 (0.43)	0.53	0.312 (0.29)
TTS medial (s)	2.84 (10.97)	2.18 (0.53)	0.67	0.242 (0.32)

*CoP, center of pressure; AP, anteroposterior; ML, mediolateral; TTS, time to stabilization*.

Data are presented as mean (95% CI). P-values and effect sizes (Cohen's d) are indicated as revealed by dependent t-tests (p <0.05). *Statistically significant; small effect: d = 0.20; medium effect: d = 0.50; large effect: d = 0.80 (Cohen, 1988).

Dependent *t*-tests revealed that the exoskeleton did not significantly influence the mean velocities and mean frequencies of the CoP signal in bipedal stance, with effect sizes being entirely small.

**Figure 3:**
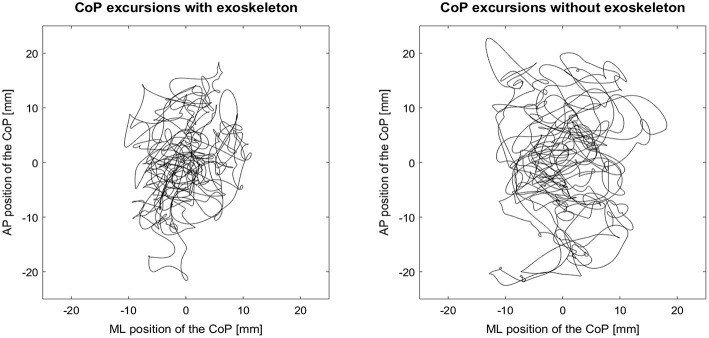
Exemplary course of the center of pressure (CoP) excursion for single-leg standing with the exoskeleton (left) and single-leg standing without the exoskeleton (right). AP, anteroposterior; ML, mediolateral.

For the single-leg stance, statistical differences between EXO and No-EXO were found. In mediolateral direction, participants showed a shorter CoP mean velocity as well as a reduced mean frequency for the EXO condition when compared to the No-EXO condition (Figure 3). These differences are reinforced by large effects sizes. Contrastingly, the exoskeleton had no impact on the dependent variables in anteroposterior direction.

**Figure 4:**
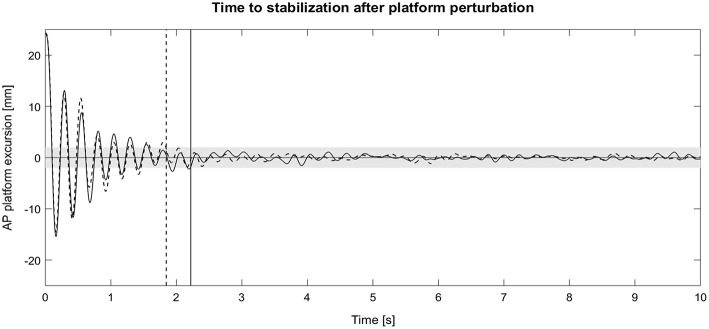
Exemplary time course of the platform excursions after anterior platform perturbation during single-leg standing with the exoskeleton (solid line) and without the exoskeleton (dashed line). Vertical lines represent the time to stabilization (TTS). AP, anteroposterior.

For the dynamic reactive balance task, the ANOVA revealed no significant main effect (*p* = 0.154) for the factor exoskeleton. Hence, the exoskeleton did not statistically affect TTS, although a large effect size ($n_{p}^{2}$ = 0.19) was indicated. *Post-hoc t*-tests, which were used to follow up on this effect size, also revealed no statistically significant difference between both exoskeleton conditions; independent of the direction of perturbation. Nonetheless, the participants tended to regain stability less quickly when wearing the exoskeleton, indicated by small to almost medium effects sizes (0.29 ≤ *d* ≤ 0.46) (Figure 4). Besides, the ANOVA indicated a significant main effect for direction of perturbation (*p* < 0.001, $n_{p\;}^{2}=$ 0.59). *Post-hoc* pairwise comparisons revealed that the TTS was significantly larger for medial perturbations as compared to anterior (*p* = 0.001) and posterior perturbations (*p* = 0.011), irrespective of the exoskeleton condition. There was no interaction effect (*p* < 0.885, $n_{p}^{2}$ = 0.02).

## Discussion

The aim of this study was to investigate if a passive unilateral LLExo has an impact on human balance control. For this purpose, we conducted two static balance tests and a dynamic reactive balance test, which were performed with and without the exoskeleton. The study yielded the following main findings: (i) the exoskeleton did not influence balance control in bipedal stance but increased mediolateral stability in single-leg stance, and (ii) the exoskeleton had no statistical effects but tended to decrease dynamic reactive balance after random platform perturbations.

### Increased Mediolateral Stability in Single-Leg Stance

The results showed that our passive unilateral LLExo did not change the spatial and frequency domains of balance control in the bipedal stance. However, the exoskeleton increased the participants' static stability in single-leg stance, indicated by a significant decrease of the CoP mean velocity in mediolateral direction. These sway reductions were accompanied by a lower mean frequency of the mediolateral component of the CoP signal, whereas in anteroposterior direction both CoP mean velocity and CoP mean frequency were not affected by the exoskeleton.

In view of these findings, our study suggests that the exoskeleton may offer the wearer some degree of mechanical stability during single-leg stance, which is reflected in the decreased mean frequency of postural sway. Interestingly, this support becomes evident in the mediolateral but not in the anteroposterior components of the CoP signal. This effect might be explained by the configuration of the exoskeleton, which permits almost unrestricted joint motion in the sagittal plane (knee flexion/extension, ankle plantarflexion/dorsiflexion) reflected in terms of neither improved nor deteriorated balance control in this plane. On the other hand, even though soft aluminum frames were used to slightly compensate for the missing DoF, it must be assumed that the exoskeleton compromised angular motions in the frontal plane (knee abduction/adduction, ankle inversion/eversion). Albeit this constraint makes balance control more difficult, it may also provide some mechanical support that gives additional passive stability to the wearer.

We believe that this support becomes evident only in static but rather unstable stance conditions, as e.g., during single-leg standing. Contrastingly, this effect seems to disappear as the balance task becomes easier, especially if the stance condition is almost mechanically stable, such as in bipedal side-by-side stance. Herein, the use of both limbs enables the subject a relatively passive control of the body's center of mass by using the hip load/unload mechanism (Winter et al., 1996). In turn, balance control under this condition does not additionally benefit from the exoskeleton.

Although literature is sparse, support for this hypothesis comes from the data previously published by Schiffman et al. (2008). The authors showed that a passive lower body prototype exoskeleton reduced postural sway excursions and maximal range of movements in 10 US army men, irrespective of whether they were carrying military loads of 20, 40, or 55 kg. These findings are also in line with the main body of research dealing with the effects of lower limb orthoses. In older adults (73 ± 6.5 years), the use of an ankle-foot orthosis was shown to reduce the CoM sway during eyes-open and eyes-closed Romberg's stance when compared to a barefoot and a shoe-alone condition (Yalla et al., 2014). Likewise, ankle-foot orthoses have been reported to enhance static postural stability measures in functional ankle instability (Hamlyn et al., 2012) and chronic stroke patients (Chern et al., 2013; Kim et al., 2015). Therefore, it seems as if both rigid braces and passive LLExos provide some kind of a bracing effect on the subject's body, ultimately increasing static postural stability. Nonetheless, as Tyson and Kent (2013) stated, the long-term effects of orthoses and exoskeletons still need to be evaluated.

### Tendency of an Impaired Dynamic Reactive Balance

While we found an increased static stability during single-leg standing and no changes with respect to bipedal standing, the exoskeleton tended to negatively affect the participants' dynamic reactive balance. Although statistically not significant, the time to stabilization in response to multidirectional platform perturbations showed a large effect sizes for the factor exoskeleton ($n_{p}^{2}$ = 0.19). Referring to this effect size, our data indicate that the exoskeleton might have impaired the capacity to compensate for these balance perturbations. It can be suggested, that the exoskeleton effectively reduced the ankle and knee range of motion in all directions of motion (Willeford et al., 2018) and by this impeded adequate compensatory postural adjustments that would be necessary to regain stability in a quick and efficient manner. This finding is highly interesting from two points of view.

First, from a neuromechanical perspective, disturbances of balance resulting in anteroposterior or mediolateral displacement of the body's center of mass with respect to the base of support are very difficult to control for the central nervous system. Adequate compensation of the disturbance requires not only sufficient muscular activity, but also call for a precise neuronal control and coordination of the skeletal muscles in temporal and spatial domains, transmitting the force to the skeleton and on to the platform in order to regain postural equilibrium after its deterioration. In single-leg stance, postural adjustments are further limited due to the small base of support and less effective postural strategies (e.g., lower overall muscle power, no use of load/unloading mechanism). Especially a loss of balance in lateral direction (medial displacement of the platform) is very difficult to recover from, because the unloaded limb is on the opposite side from the direction of fall, whereas in the A/P directions a corrective step forward or backward is possible (Winter et al., 1996). This can be seen from the comparisons between the different directions of perturbation, which indicated that the time to stabilization was significantly larger for medial perturbations as compared to the anterior and posterior perturbations.

Secondly, our results are relevant from a socio-economic and clinical perspective. Research has shown that the risk of falling is more closely related to dynamic stability than static stability (Rubenstein, 2006). Beyond that, most fall-related events occur under dynamic conditions (Blake et al., 1988). Given the dynamic nature of most activities of daily living along with the human's susceptibility to falls, the augmentation or preservation of dynamic balance is a particular issue of concern. Taking into account our results, it is indicated that passive and rigid elements attached to the lower extremities could decrease dynamic reactive balance, which emphasizes that balance controllers (Emmens et al., 2018) and active components (e.g., compliant actuators; Cestari et al., 2017) would be necessary to compensate for the potential disadvantages that might accompany with the application of exoskeletons to the human body. The benefit of such systems has been reported by Emmens et al. (2018), who showed that a powered ankle-foot orthosis with a body sway based controlled can assist humans in maintaining balance when counteracting anteroposterior perturbations. Therefore, predictions of upcoming disturbances (Chen et al., 2018) and early detection of postural instability (Chang et al., 2017), concomitant with a profound understanding about the human-exoskeleton interaction and its impact on balance control are an essential prerequisite in order to assist or augment human motor control and balance.

To the best of our knowledge, the results presented here are the first addressing the impact of a passive LLExo on dynamic reactive balance. Previously, mainly the effects of passive ankle-foot orthoses or prophylactic ankle braces on functional reach in the star excursion balance test have been examined. Those studies showed that dynamic balance did not differ among different orthotic conditions in healthy people (Hadadi et al., 2014; Willeford et al., 2018), however that in ankle instability patients functional reach distance can be significantly increased when compared to no-orthosis condition (Hadadi et al., 2014; Crockett and Sandrey, 2015; Abbasi et al., 2019). Contradictory results are reported for dynamic proactive balance. While a study by Maeda et al. (2016) revealed that a semi-rigid brace acutely enhanced dynamic balance by decreasing the dynamic postural stability index after single-leg landing, Hueber et al. (2017) did not find ankle bracing to substantially affect body mechanics during landing. With respect to dynamic reactive balance, which might be the most complex balance task, there was only one study available. Cikajlo et al. (2016) observed acute improvements in postural responses following perturbations at the pelvis level when suitable ankle-foot ortheses were applied. Using principal component analysis, Tsai et al. (2018) showed that the application of bilateral ankle-foot orthoses with mechanical ankle constraints can lead to changes in the coordination solution, with increasing reliance on compensatory knee movements. However, as we used an exoskeleton covering the whole limb, compensation at the knee joint level are not or only partially possible so that compensatory motions of proximal body segments and joint come into question.

Hence, the design of the exoskeleton along with the complexity of the balance task used in our study might be responsible for the results deviating from the literature. At the same time, it needs to be mentioned that we did not assess long-term effects and that the adaptations found might disappear after long-term use of the exoskeleton. It is well-known, that postural synergies change with continuous balance training (Serrien et al., 2017) as well as with repeated exposure to orthotic devices (Tyson and Kent, 2013; Cikajlo et al., 2016).

### Study Limitations

There are some study limitations, which need to be considered when discussing our results. We used a passive LLExo that was applied to the left leg of the participants. This minimal setup was chosen to get a fundamental knowledge about the effects of exoskeletons on the human body and motor control. Although exoskeletons are thought to actively support the wearer, investigations of the influence of a passive exoskeleton is of importance as well, e.g., when the robotic exoskeleton runs out of power or no support is desired. To overcome the shortcoming of not having a bilateral exoskeleton, we conducted both bipedal and unilateral balance tests. Nevertheless, it remains to be investigated whether the application of a bilateral exoskeleton equalizes or amplifies the above-depicted effects. Another aspect that should not be ignored is that our exoskeleton had only one DoF in the knee and ankle joint, respectively. This may have strongly influenced the balance control strategies of our participants. Albeit the ankle strategy is well-known to be the major control strategy used for anteroposterior balance control, in single-leg stance the ankle also contributes to the regulation of mediolateral sway. Therefore, future studies should consider the application of exoskeletons with two or three DoF at the ankle joint. Beyond that, segment length adjustments of the exoskeleton were not possible at this point. Hence, only subjects with fitting lower limb segment length and shoe size could participate in this study, which has led to the small and homogeneous study sample. In consequence, the probability of type II error is considerably increased and especially the assumptions about dynamic balance impairments must be treated with caution. Furthermore, due to the homogenous sample and the specificity of the exoskeleton, a generalization of our results is not possible. All these points will be addressed in the further development of the exoskeleton.

## Conclusion

Bearing in mind these limitations, the present study has shown that our passive unilateral LLExo improved static stability but tended to decrease dynamic reactive stability, each in single-leg stance. Considering the mean frequency of postural sway, it seems that the exoskeleton provides some passive mechanical stability under static conditions, whereas under dynamic conditions the exoskeleton might inhibit adequate postural adjustments resulting in a prolonged time to stabilization. In contrast, the exoskeleton did not influence bipedal balance control. Obviously, the contralateral (right) leg countered any stabilizing or destabilizing effects of the exoskeleton.

Therefore, our study provides important findings on human-exoskeleton interaction with strong implications for the future design and application of exoskeletons. Specifically, the study highlights the significance of taking into account the control mechanisms and strategies of human posture and locomotion in order to develop exoskeletons that are able to relieve or to augment its wearer as well as to be used for fall prevention in the elderly.

## Data Availability

The datasets generated for this study are available on request to the corresponding author.

## Ethics Statement

This studies involving human participants were reviewed and approved by ethics committee of the Karlsruhe Institute of Technology. The patients/participants provided their written informed consent to participate in this study.

## Author Contributions

SR, IP, JB, TA, and TS: conceptualization, methodology, software, and writing—review and editing. SR, IP, and JB: data curation and visualization. SR: formal analysis, writing—original draft preparation, and validation. SR and IP: investigation. TA and TS: project administration, resources, and supervision.

### Conflict of Interest Statement

The authors declare that the research was conducted in the absence of any commercial or financial relationships that could be construed as a potential conflict of interest.
